# Incidence and risk factors of early HCC occurrence in HCV patients treated with direct acting antivirals: a prospective multicentre study

**DOI:** 10.1186/s12967-019-2033-x

**Published:** 2019-08-28

**Authors:** Luca Rinaldi, Alessandro Perrella, Maria Guarino, Massimo De Luca, Guido Piai, Nicola Coppola, Pia Clara Pafundi, Fortunato Ciardiello, Morena Fasano, Erika Martinelli, Giovanna Valente, Riccardo Nevola, Caterina Monari, Lucia Miglioresi, Barbara Guerrera, Massimiliano Berretta, Ferdinando Carlo Sasso, Filomena Morisco, Antonio Izzi, Luigi Elio Adinolfi

**Affiliations:** 1 Department of Advanced Medical and Surgical Sciences, University of Campania “Luigi Vanvitelli”, Naples, Italy; 2 Immunological and Neurological Infectious Diseases Unit, Cotugno Hospital, Naples, Italy; 3 Department of Clinical Medicine and Surgery, Gastroenterology Unit, Federico II University, Naples, Italy; 4 Hepatology and Pancreas Unit, Cardarelli Hospital, Naples, Italy; 5 Liver Diseases and Transplant Unit, Department of Medical Sciences, S. Anna and S. Sebastiano Hospital, Caserta, Italy; 6 Department of Precision Medicine, University of Campania “Luigi Vanvitelli”, Naples, Italy; 7 Medical Unit, New Hospital of Marcianise, Marcianise, CE Italy; 8 Department of Medical Oncology, Centro di Riferimento Oncologico, Istituto Nazionale Tumori Aviano, Aviano, PN Italy

**Keywords:** HCC, Direct acting antivirals, HCV cirrhosis, Immune-surveillance

## Abstract

**Background:**

An unexpected increased HCC recurrence and occurrence rate among HCV patients treated with direct acting antivirals combination has been reported. Aim of the study was the evaluation of early HCC occurrence rate and its risk factors in a HCV infected population, treated with direct-acting-antivirals.

**Methods:**

According to the Italian ministerial guidelines for direct-acting-antivirals treatment, 1022 consecutive HCV patients treated with direct-acting-antivirals were enrolled. Patients either with active HCC at imaging or history of previous treated HCC, HBV or HIV co-infection, or liver transplant recipients were excluded. The SVR, defined as the persistent absence of detectable serum HCV-RNA 12 weeks after the end of treatment (SVR12), was assessed for all enrolled patients. Abdominal ultrasound was performed before starting antiviral therapy, and repeated every 6 months. HCC was diagnosed according to the international guidelines. Patients showing either nodular patterns suggestive of HCC or with uncertain dynamic vascular behaviour were excluded from a further follow-up.

**Results:**

Nine hundred and eighty-five patients completed the 48 weeks follow-up after the end of treatment. A Sofosbuvir-based regimen was administered in the 74.9% of patients, among whom, the 71.6% underwent a simultaneous Ribavirin administration. A sustained virological response at 12 weeks off treatment was documented in 966 patients (98.2%). During the post treatment follow-up HCC was detected in 35 patients, with a cumulative incidence rate of the 3.55%. At multivariate analysis, four variables resulted independently associated with HCC development, both in a cirrhosis based and a class B Child based model, respectively: cirrhosis/class B Child, therapeutic schedule including Sofosbuvir without Ribavirin, liver stiffness values, male gender and presence of diabetes. A multivariate analysis performed on Child A cirrhotic patients, showed that Sofosbuvir based therapeutic treatment without Ribavirin had a HCC occurrence 5.7 higher than Ribavirin-based schedules with or without Sofosbuvir (p < 0.0001, OR: 5.686, 95% CI 2.455–13.169).

**Conclusions:**

Our data suggest that early HCC occurrence appears more frequently related to Sofosbuvir-based therapy without Ribavirin which, indeed, seems to play a protective role on HCC onset. Therefore, a careful follow-up should be mandatory, especially in those regimens including Sofosbuvir without Ribavirin.

## Background

Direct acting antiviral (DAA) based treatment dramatically changed the natural history of hepatitis C virus (HCV) chronic infection. In fact, whereas previous antiviral regimens were characterized by a sustained virological response (SVR) rate of the 55–65%, these new therapies schedules, according to several clinical trials and the available real practice reports, reached more than 90% of SVR [1]. Indeed, the viral clearance embodies a fundamental turning point in the chronic HCV infection, as it may prevent the most part of severe complications and disease evolution of persistent infection, since these are related to immune system imbalance because of viral replication [2, 3].

New DAAs schedule activity on immune system is still unknown. However, DAA treatment seems to exert a certain effect on immune response [4, 5].

Hepatocellular carcinoma (HCC) represents one of the most important complications of chronic liver disease, with an incidence rate of the 1–6% per year, and is mainly related to two well-known pathways: (a) to fibrosis due to continuous necrosis and (b) to immune-surveillance failures attributable to persistent viral replication with immune system escape mechanisms [6]. A direct carcinogenic effect of HCV proteins, which deregulate host cell cycle checkpoints and the virus and immune-mediated oxidative stress, thus leading to DNA mutations in liver cells, is also known [7]. Therefore, according to all these evidences, HCV eradication and its necro-inflammation activity may be crucial in HCC prevention.

So far, several long-term studies on patients treated with Interferon and Ribavirin-based regimens globally reported a reduced HCC incidence in about the 75% of patients achieving a SVR, and a residual risk of HCC development, mainly associated with comorbidities, such as metabolic syndrome and type 2 diabetes [8, 9].

Recently, an unexpected increased HCC recurrence and occurrence rate among HCV patients treated with DAAs combination has been reported, raising some concerns about a possible indirect role of the new antiviral drugs [10–13]. Conversely, other studies showed either a stable or a decreased overall HCC incidence [14, 15].

These controversial data are triggering an interesting debate on the residual risk of HCC development after DAAs treatment, especially about the possible involvement of DAAs in liver carcinogenesis.

According to the aforementioned evidences [7–9], our study assessed DAAs efficacy and early HCC occurrence rate and risk factors in a real life setting on a well-studied HCV population regularly followed during and after an antiviral treatment with DAAs.

## Patients and methods

### Study design and patients population

From February 2015 to February 2017 a group of 6 Hospital and Academic Centres in Southern Italy (Campania Region) conducted a prospective, real-life study on efficacy of DAAs treatment schedule. 1022 consecutive HCV patients treated with IFN-free DAAs regimens were enrolled.

According to the Italian ministerial guidelines for DAAs treatment, inclusion criteria were HCV-RNA serum positivity and fibrosis stage ≥ F3 according to Metavir score (the Italian reimbursement criteria were applicable only for patients with F3–F4 fibrosis), assessed either by liver biopsy or transient elastography (TE). The TE was performed by Fibroscan^®^ (Echosens, Paris, France), according to standard criteria.

In this real life population study, patients with either active HCC at imaging or history of previous treated HCC, HBV or HIV co-infection, or liver transplant recipients were excluded.

The baseline HCC screening for all patients enrolled in our cohort was performed according to the European Association for the Study of Liver (EASL) guidelines [16].

An abdominal ultrasound (US) was performed before starting the antiviral therapy (within 1 month). Each US was performed by an experienced operator [more than 5000 exams performed following SIUMB (Italian Society of Ultrasound Medicine) certification], to minimize the bias of a multicenter, unmonitored study. Either a contrast-enhanced ultrasonography (CEUS), a dynamic computed tomography (CT) scan or dynamic magnetic resonance imaging (MRI) was performed to characterize incidental hepatic lesions. Patients showing either nodular patterns suggestive of HCC or with uncertain dynamic vascular behaviour at treatment enrolment were instead excluded from a further follow-up.

HCV-RNA was assessed by real-time PCR (COBAS^®^ TaqMan, AmpliPrep, Roche), with a detection limit of 15 IU/mL.

Cirrhosis diagnosis was based on clinical, biochemical, ultrasonographic, elastographic and, where available, histological parameters. In particular, among cirrhotic patients, liver function was graded according to the Child–Turcotte–Pugh (CTP) score system.

**Fig. 1:**
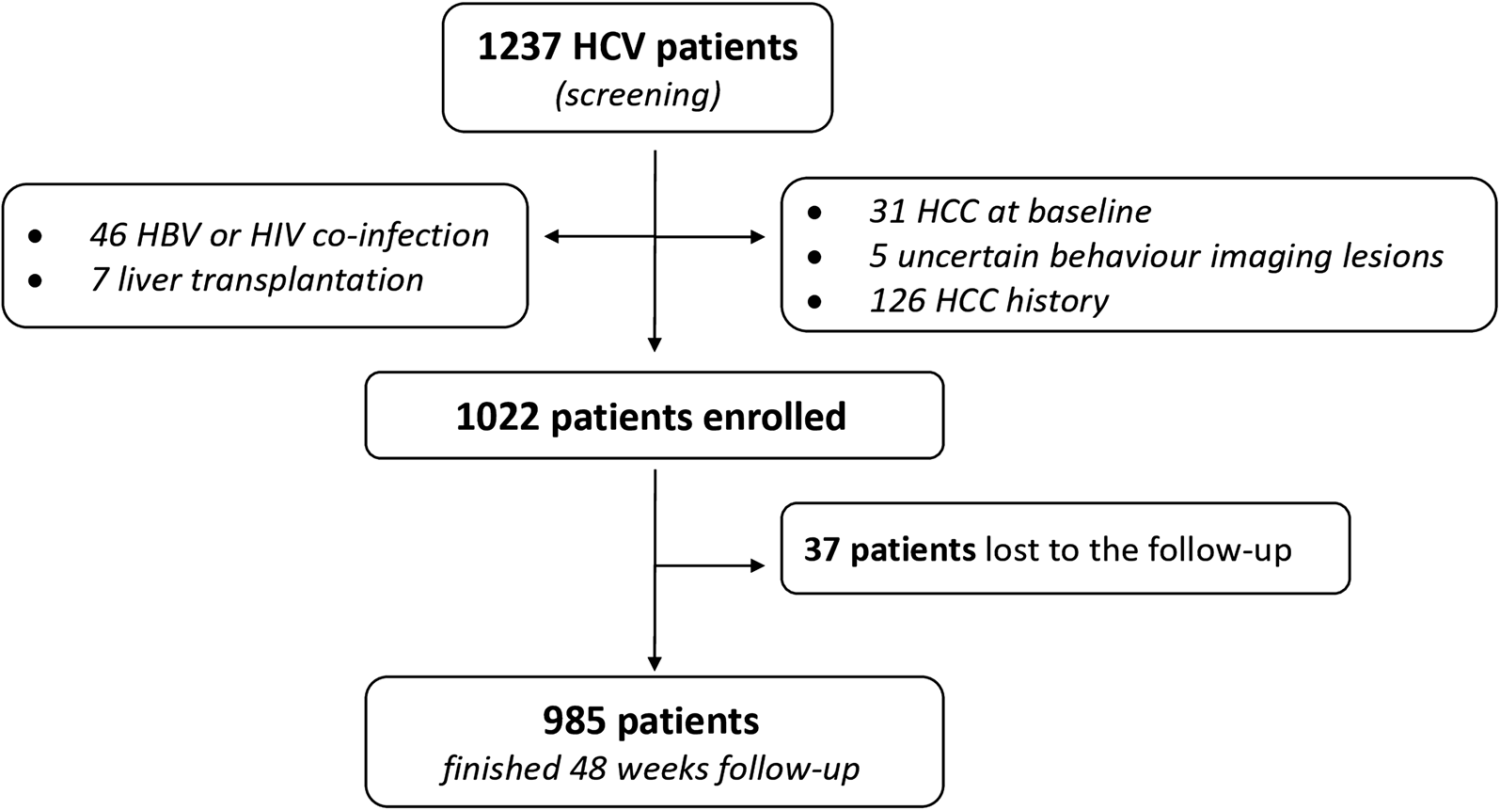
Study flow

Demographic characteristics and clinical parameters at baseline, were recorded and reported (Fig. 1).

The study was performed according to the 1976 Declaration of Helsinki and its later amendments and approved by our local Ethic Committee. All patients gave their informed consent to the study.

### Antiviral treatment

Patients eligibility to HCV treatment with IFN-free DAAs regimens was assessed following the priority criteria established in February 2015 by the National Scientific Society and Registry of the Italian Medicines Agency Committee (AIFA). The prescribing clinicians chose the treatment regimen in accordance with the National and International Guidelines and its most recent updates at that time [17, 18].

The treatment duration (12/24 weeks) was established based on the severity of liver disease, with a longer treatment reserved to cirrhotic patients.

Patients were treated either with Sofosbuvir + Ribavirin (SOF/RBV-dual), Simeprevir + Sofosbuvir ± Ribavirin, Daclatasvir + Sofosbuvir ± Ribavirin, Ledipasvir + Sofosbuvir ± Ribavirin or Ombitasvir/Paritaprevir/Ritonavir (2D) ± Dasabuvir (3D) ± Ribavirin.

Successively, therapeutic regimens were categorized as follows: Sofosbuvir (SOF)-based, Ribavirin (RBV)-included and all treatment Sofosbuvir + Ribavirin based (SOF/RBV-all). Ribavirin dosage was never reduced (according to adverse events) below 600 mg day in all therapeutic schedules.

### Patients follow-up

Nine hundred and eighty-five (n = 985) of the 1022 enrolled patients completed the 48 weeks follow-up after the end of treatment. Thirty-five patients were excluded from the analysis due to incomplete follow-up data, whilst three died during the antiviral therapy (due to causes unrelated to DAAs).

Virological response to the therapy was assessed by real-time PCR, with HCV-RNA detection at the end of the treatment, 12 and 24 weeks after its end. The SVR, defined as the persistent absence of detectable serum HCV-RNA 12 weeks after the end of treatment (SVR12), was assessed for all enrolled patients. Any relapse of serum HCV-RNA during follow-up was also recorded.

At least three ultrasound examinations were performed on every enrolled patient during the established follow-up period (before starting therapy and every 6 months), according to the HCC surveillance program and the study design. Any detected liver lesion was evaluated by imaging technique workup (CEUS, or dynamic CT scan or dynamic MRI) according to EASL guidelines [16].

The diagnosed HCC were recorded and scored according to Barcelona Clinic Liver Cancer (BCLC) staging system [19]. Once a patient was diagnosed with HCC, the follow-up was discontinued.

### Endpoints of study

Primary endpoint was the assessment of HCC occurrence rate in HCV patients within 48 weeks after DAAs-IFN free treatment, whilst secondary endpoint was the evaluation of risk factors associated with HCC occurrence.

As an additional end-point, a sub-analysis exclusively focused on cirrhotic patients was performed, to reduce any possible selection bias. Similarly, an additional sub-analysis within cirrhotic patients focused on Child A or Child B patients, most of all to obtain an indication to the prescription of 3D–2D therapies only in Child A patients.

### Statistical analysis

Baseline characteristics and measures of clinical and demographic variables were expressed as absolute and percentage (for categorical variables) or median and interquartile range (IQR) for continuous variables. Categorical data were compared using either the Pearson Chi-square test (with Mantel–Haenszel common odds ratio estimate) or Fisher Exact test when indicated, and continuous variables by non-parametric Mann–Whitney U test or Kruskal–Wallis test, where indicated. A logistic binary regression model (backward step-wise model) was performed to evaluate independent factors associated with HCC occurrence. Moreover, a stratification analysis was also performed, in order to better assess the much higher HCC occurrence with respect to duration of therapy.

To avoid a collinearity bias, the presence of cirrhosis and the class B CTP were evaluated separately in two different models. For all statistical comparisons, a two-tailed significance level of 0.05 was used, with a 95% confidence interval. All analyses were performed using SPSS software, version 24.0 (IBM SPSS Statistics for Windows, Version 24.0. Armonk, NY:IBM Corp.).

## Results

Nine hundred and eighty-five patients completed treatment and follow-up, according to the study design, from February 2015 to February 2017. Data were equally distributed among the participant Centres. The baseline demographic characteristics of the enrolled population are reported in Tables 1 and 2.

**Table 1:** Overall baseline characteristics according to SVR status

Parameters	Overall (n = 985)	SVR (n = 966)	No SVR (n = 19)
Sex, n (%)			
M	543 (55.1)	529 (54.8)	14 (73.7)
F	442 (44.9)	437 (45.2)	5 (26.3)
Age, years, median [IQR]	67 [59–73]	67 [59–73]	66 [60–71]
BMI, kg/m^2^, median [IQR]	26 [24–28.4]	26 [24–28.3]	27 [23.3–30.6]
Diabetes, n (%)	131 (13.3)	129 (13.4)	2 (10.5)
Metabolic syndrome, n (%)	69 (8)	69 (8.1)	–
Liver disease stage, n (%)			
Advanced fibrosis	254 (25.8)	252 (26.1)	2 (10.5)
Cirrhosis	731 (74.2)	714 (73.9)	17 (89.5)
HCV genotype, n (%)			
1	765 (77.7)	754 (78.1)	12 (63.2)
2	160 (16.2)	155 (16.1)	4 (21.1)
3	50 (5.1)	47 (4.9)	3 (15.8)
4	10 (1.0)	10 (1.0)	0
Liver stiffness, kPa, median [IQR]	17.3 [11.9–35.3]	16 [11.8–23]	23.4 [14.6–41.7]
Treatment duration, n (%) (weeks)			
12	612 (62.1)	603 (62.4)	9 (47.4)
24	373 (37.9)	363 (37.6)	10 (52.6)
SVR12, n (%)	966 (98.1)		
DAAs regimen, n (%)			
SOF/RBV (dual)	417 (42.3)	408 (42.2)	10 (52.6)
SOF/LDV ± RBV	183 (18.6)	186 (19.3)	3 (15.8)
SOF/DCV ± RBV	39 (4.0)	33 (3.4)	0 (0.0)
SOF/SIM ± RBV	99 (10.1)	97 (10)	2 (10.5)
2D/3D ± RBV	247 (25.0)	242 (25.1)	4 (21.1)
Treatment classes, n (%)			
SOF based	738 (74.9)	723 (74.8)	15 (78.9)
RBV included	705 (71.6)	693 (71.7)	12 (63.2)
SOF/RBV (all)	573 (58.2)	561 (58.1)	12 (63.2)
HCC occurrence, n (%)	35 (3.6)	35 (3.6)	0 (0)

**Table 2:** Overall baseline characteristics according to liver disease stage

Parameters	Cirrhosis (n = 731)	Advanced liver fibrosis (n = 254)
Sex, n (%)		
M	391 (53.5)	152 (59.8)
F	340 (46.5)	102 (40.2)
Age, years, median [IQR]	68 [60–74]	66 [58–72]
BMI, kg/m^2^, median [IQR]	26 [23.9–28.4]	26.1 [24.1–28.3]
Diabetes, n (%)	99 (13.6)	32 (12.6)
Metabolic syndrome, n (%)	53 (8.2)	16 (7.5)
Child–Turcotte–Pugh, n (%)		
Class *A*	649 (88.8)	–
Class *B*	82 (11.2)	–
HCV genotype, n (%)		
1	561 (76.7)	205 (80.7)
2	121 (16.6)	38 (15)
3	43 (5.9)	7 (2.8)
4	6 (0.8)	4 (1.6)
Liver stiffness, kPa, median [IQR]	21 [15.6–27.2]	10.7 [10–11.8]
Treatment duration, n (%) (weeks)		
12	401 (54.9)	211 (83.1)
24	330 (45.1)	43 (16.9)
SVR12, n (%)	714 (97.7)	252 (99.2)
DAAs regimen, n (%)		
SOF/RBV	322 (44)	96 (37.8)
SOF/LDV ± RBV	158 (21.6)	31 (12.2)
SOF/DCV ± RBV	26 (3.6)	7 (2.8)
SOF/SIM ± RBV	78 (10.7)	21 (8.3)
2D/3D ± RBV	147 (20.1)	109 (39)
Treatment classes, n (%)		
SOF based	584 (79.9)	154 (60.6)
RBV included	549 (75.1)	156 (24.2)
SOF/RBV	450 (61.6)	123 (48.4)
HCC occurrence, n (%)	35 (4.8)	0 (0)

Within the subgroup of cirrhotic patients (n = 731), four hundred and eighty-five (74.8%) CTP A and 64 (78%) of the CTP B patients underwent a RBV scheduled treatment. An overall successful treatment outcome, with SVR achievement, was obtained in 966 patients (98.1%).

During the post treatment follow-up, HCC onset was reported in 35 patients, with a cumulative incidence rate of the 3.55%, which raised till the 4.65% considering only the cirrhotic subset of patients. Two HCC cases were recorded at the end of treatment, 16 cases 12–24 weeks after treatment, and 17 cases 36–48 weeks after treatment.

All patients with HCC occurrence did not show any viral relapse, achieving a SVR. The median diameter of lesions was 33 mm (range 18–57 mm). None of the patients with HCC was an active alcohol consumer, whilst three subjects (8.5%) were smokers (about 10 cigarettes/day).

According to BCLC classification, patients were stratified as follows: 27 patients as stage A, 4 patients as stage B, 4 patients as stage C. Among HCC patients, 31 of them (88.5%) underwent a SOF-based treatment and 19 (61.3%) were treated without RBV.

Based on univariate analysis, gender (p = 0.048), age (p = 0.045), CTP B stage (p = 0.001), presence of diabetes as comorbidity (p = 0.007), presence of cirrhosis (p = 0.002), and liver stiffness value (p < 0.0001) were significantly associated with HCC occurrence. A SOF-based therapeutic regimen without RBV was significantly associated with HCC development (p = 0.003). Conversely, the use of Ribavirin was associated with a reduced risk of HCC development (p < 0.0001), regardless of the DAAs regimen (Table 3).

**Table 3:** Clinical and demographic characteristics in patients with and without HCC occurrence—univariate and multivariate analysis

Parameters	Univariate analysis			Multivariate analysis (cirrhosis)		Multivariate analysis (Child–Pugh)	
	Patients without HCC (n = 950)	Patients with HCC (n = 35)	p	O.R. [95% C.I.]	p	O.R. [95% C.I.]	p
Male gender, n (%)	518 (54.5)	25 (71.4)	0.048	2.852 [1.162–6.999]	0.022	2.738 [1.109–6.762]	0.029
Age, years, median [IQR]	67 [59–73]	69 [64–74]	0.045				
BMI, kg/m^2^, median [IQR]	26 [24–28.4]	25.4 [23.4–28]	0.17				
Presence of diabetes, n (%)	121 (12.8)	10 (28.6)	0.007	0.392 [0.155–0.988]	0.047	0.369 [0.146–0.931]	0.047
Presence of cirrhosis, n (%)	696 (73.4)	35 (100)	0.002	0.153 [0.019–1.239]	0.079		
CTP B class^a^ n (%)	72/696 (10.3)	10/34 (29.4)	0.001			0.430 [0.170–1.086]	0.074
HCV genotype 1, n (%)	724 (77.3)	29 (82.9)	0.44				
24 weeks treatment duration, n (%)	354 (37.3)	19 (54.3)	0.041				
Baseline HCV-RNA, UI/ml, median [IQR]	995,500 [350,000–2,646,500]	729,000 [290,000–1,238,000]	0.123				
Liver stiffness, kPa, median [IQR]	14.9 [11.8–22]	32 [18.5–44.4]	< 0.0001	1.048 [1.020–1.077]	0.001	1.057 [1.031–1.084]	0.000
SVR12, n (%)	933 (98.2)	33 (94.3)	0.09				
DAA-SOF based, n (%)	707 (74.6)	31 (88.6)	0.09				
DAA-RBV included, n (%)	690 (72.8)	15 (42.9)	< 0.0001				
DAA-SOF + RBV, n (%)	562 (59.2)	12 (34.3)	0.003				
DAA-SOF without RBV, n (%)	145 (20.5)	19 (61.3)	0.003	15.363 [6.668–35.396]	0.000	0.059 [0.025–0.137]	0.000

^a^Computed based on number of cirrhotic patients

Moreover, the duration of therapy also resulted significantly associated with HCC occurrence (p = 0.041). Nevertheless, the greatest percentage of HCC occurrence in the group 24 weeks (5.1%) versus the group 12 weeks (2.6%) suggested us to perform a stratified analysis for each group of duration therapy. The 24 weeks group underwent more SOF therapy than the 12 weeks group (96.7% vs 61.8%). However, a stratification for duration of therapy (12 vs 24 weeks) and type of treatment (SOF-without-RBV vs all other Tx) showed a statistically significant association between that variable and HCC onset in both group: 12 weeks group (11.3% vs 1.3%, p = 0.001) and 24 weeks group (12.3% vs 3.1%, p = 0.005), independently from the duration of treatment.

We then performed two different multivariate models, in order to assess separately the presence of cirrhosis and the class B CTP, in order to avoid a collinearity bias.

The first model, built based on the presence of cirrhosis, showed four variables resulted independently associated with HCC development: the use of a SOF-based regimen without RBV (p = 0.000, O.R.: 15.363, 95% C.I. 6.668–35.396), liver stiffness values (p = 0.001, O.R.: 1.048, 95% C.I. 1.020–1.077), male gender (p = 0.022, O.R.: 2.852, 95% C.I. 1.162–6.999) and presence of diabetes as comorbidity (p = 0.047, O.R.: 0.392, 95% C.I. 0.155–0.988), whilst presence of cirrhosis just showed a trend at multivariate analysis, without reaching the statistical significance (p = 0.079; O.R.: 0.153; 95% C.I. 0.019–1.239) (Table 3).

The second model, built instead considering the CTP class B, similarly showed the same four variables as independently associated with HCC development: the use of a SOF-based regimen without RBV (p = 0.000, O.R.: 0.059, 95% C.I. 0.025–0.137), liver stiffness values (p = 0.000, O.R.: 1.057, 95% C.I. 1.031–1.084), male gender (p = 0.029, O.R.: 2.738, 95% C.I. 1.109–6.762) and presence of diabetes as comorbidity (p = 0.047, O.R.: 0.369, 95% C.I. 0.146–0.931), whilst presence of CTP class B just showed a trend at multivariate analysis, without reaching the statistical significance (p = 0.074; O.R.: 0.430; 95% C.I. 0.170–1.086) (Table 3).

A further sub-analysis, focusing only on cirrhotic patients was performed. In this subset of patients, at univariate analysis (Table 4) male gender (p = 0.04), presence of diabetes (p = 0.006), CTP class B (p = 0.001), liver stiffness ≥ 20 kPa (p = 0.01), and the use of SOF-based regimen without RBV (p < 0.0001), were significantly associated with HCC occurrence. At multivariate analysis, four variables resulted independently associated with HCC development: the use of a SOF-based regimen without RBV (p < 0.0001, OR: 6.145, 95% CI 2.97–12.73), CTP class B (p = 0.004, OR: 3.29, 95% CI 1.46–7.44), male gender (p = 0.019, OR: 2.58, 95% CI 1.17–5.69) and presence of diabetes as comorbidity (p = 0.043, OR: 2.3, 95% CI 1.03–5.16) (Table 5).

**Table 4:** Clinical and demographic characteristics in cirrhotic patients subgroup with and without HCC occurrence (N = 731)—univariate analysis

Variables	Cirrhotic patients without HCC (n = 696)	Cirrhotic patients with HCC (n = 35)	p
Male gender, n (%)	367 (52.7)	24 (70.6)	0.04
Age, years, ≥ 67, n (%)	373 (53.6)	23 (67.6)	0.11
BMI, kg/m^2^ ≥ 26, n (%)	285 (51.4)	14 (42.4)	0.32
Presence of diabetes, n (%)	89 (12.8)	10 (29.4)	0.006
CTP B class, n (%)	72 (10.3)	10 (29.4)	0.001
HCV genotype 1, n (%)	524 (76.2)	28 (82.4)	0.41
24 weeks treatment duration, n (%)	311 (44.6)	19 (55.9)	0.19
Baseline HCV-RNA, UI/ml, ≥ 1 × 10^6^, n (%)	381 (54.7)	13 (38.2)	0.06
Liver stiffness, kPa ≥ 20, n (%)	176 (49.7)	24 (72.7)	0.01
SVR12, n (%)	682 (97.8)	32 (94.1)	0.16
DAA-SOF based, n (%)	555 (79.6)	30 (88.2)	0.31
DAA-RBV included, n (%)	535 (76.8)	15 (44.1)	< 0.0001
DAA-SOF + RBV, n (%)	438 (63.0)	12 (35.3)	0.001
DAA-SOF without RBV, n (%)	113 (16.2)	18 (52.9)	< 0.0001

**Table 5:** Multivariable logistic regression analysis of risk factors for HCC occurrence in the cirrhotic patients subgroup (N = 731)

Variables associated with HCC	Beta coefficient	p-value	Odds ratio	C.I. 95%	
				Low	Upper
SOF without RBV	1.82	< 0.0001	6.145	2.97	12.73
Child–Turcotte–Pugh B class	1.19	0.004	3.29	1.46	7.44
Male gender	0.95	0.019	2.58	1.17	5.69
Presence of diabetes	0.83	0.043	2.30	1.03	5.16

Subsequently, we analysed HCC occurrence only in the 649 CTP class A cirrhotic patients, according to the therapeutic schedule. The multivariate analysis showed that SOF-based therapeutic treatment without Ribavirin had a HCC occurrence 5.7 higher than with Ribavirin in the schedule or compared to schedules without Sofosbuvir (2D–3D based therapy) (Table 6).

**Table 6:** Multivariable logistic regression analysis of risk factors for HCC occurrence in the Child–Turcotte–Pugh Class A subgroup (N = 649)

Variables associated with HCC	Beta coefficient	p-value	Odds ratio	C.I. 95%	
				Low	Upper
Male gender	1.166	0.017	3.210	1.237	8.332
SOF without RBV	1.738	< 0.0001	5.686	2.455	13.169

A logistic regression was used to compare multiple causal variables on HCC development, also correcting for the potential confounding effects.

Based on our findings, in order to avoid any possible bias, we established a further univariate statistical analysis on patients undergoing a 2D–3D treatment schedule, verifying the presence of any difference with respect to patients who had undergone SOF-based antiviral regimens, with respect to gender, CTP class, albumin serum level, bilirubin, INR, diabetes, liver stiffness and platelets. According to our results, we did not find any significant statistical difference in our real life population.

## Discussion

Hepatitis C virus pathogenesis and its natural history are characterized by several factors, among which those implicating immune system activity. This, in fact, involves regulatory and effector environments, since the acute phase of infection plays an important role [20]. In this latter case, the chronic infection underlies a persistent activity of the immune system, with a specific cytokines environment leading first to a liver necro-inflammation, and then to HCC onset (which may still occur over the years, as previously suggested) [21]. HCC onset, in particular, was recently related to a persistent inflammatory network mainly supported by IL-17, TGF-beta and deactivation of T Regulatory cells [22]. Based on these findings, one of the most important purposes of the antiviral treatment in persistent infection is represented by the virus eradication in order to prevent HCC occurrence.

Previously, in interferon-based regimens, the reduction of HCC incidence, but not its disappearance, had been associated with several factors, among which: the cirrhotic persistence stage, advanced age, presence of latent HCV mutations, presence of comorbidities such as diabetes [8, 9]. The approval of second wave DAA represents a recent event and still does not allow for a long-term assessment of SVR impact on HCC incidence [23].

Recently, two interesting studies have suggested some mechanisms as possible HCC inducers after DAA treatment. Debes et al. [24] identified, in patients who developed HCC de novo after DAA treatment, an higher value of 9 inflammatory cytokines, measured in serum before treatment (MIG, IL22, TRAIL, APRIL, VEGF, IL3, TWEAK, SCF, IL21), assuming a possible role in carcinogenesis.

Faillaci et al. [25] showed that the DAAs-mediated increase of VEGF favors HCC recurrence/occurrence in susceptible patients, i.e. who already have abnormal activation of neo-angiogenetic pathways in liver tissues, as showed by an increase in angiopoietin-2, studied in neoplastic and cirrhotic tissue.

This independent, real life study showed that the 61% of patients who developed a HCC had either a single small or 2–3 small nodules, while the remaining 39% were diagnosed with larger and infiltrative cancers. According to our data, early HCC occurrence seems to be associated with to well-known factors like diabetes, liver stiffness and advanced CTP stage. Furthermore, we also found that second wave HCV DAA therapy without a contemporary RBV treatment also seems to be related to an early HCC onset. Particularly, and more interestingly, this association with neoplasms onset seems strongly occurring among CTP A patients, hence suggesting that fibrosis might not represent the only factor possibly associated with HCC occurrence in this setting of patients.

Patients who showed HCC onset achieved SVR. Our data, according to previous findings, while proving that DAAs treatment does not increase the overall risk of HCC, also appear to demonstrate that the successful antiviral therapy is not able to prevent HCC onset even though patients achieve SVR [26]. More interestingly, this latter scenario, upon a multivariate analysis, seems to be more frequently related to SOF based regimens without Ribavirin, whose use, conversely, appears associated with a reduced HCC risk.

These data could be in part explained with the well-known immune-modulating activity of RBV, independently from the dosage, which may improve immune surveillance, while both boosting Th1 response and switching from Th2 to a Th1 CD4 pattern too [27]. Moreover, some kind of protective activity of RBV against HCC, when associated with Doxorubicin, most likely implicating the immune-surveillance, has also been recently demonstrated [28].

It could also be speculated that a rapid reduction in the HCV viral load may impact the tumor immunity, since it might affect the balance between pro-tumor and anti-tumor immune functions previously reported [29].

Nonetheless, despite this scientific evidence and the possible suggestion of a pathogenetic model, the related fine mechanisms still remain unclear and are most likely associated with several factors.

Further mechanisms that could explain these events may be related to the mitochondrial dysregulation these new drugs may induce, even though literature data regarding this issue are contradictory [30, 31].

Consistently with our data, the duration of therapy did not result significantly associated with HCC occurrence. Though a much higher percentage of HCC occurrence in the 24 weeks group versus the 12 weeks group, the stratification for duration of therapy did not affect the statistically significance between HCC onset and type of treatment.

It could be argued that our findings may be related to HCC micro nodules already placed in the liver before treatment, since the majority of nodules are bigger than 2 cm, as such rapid HCC growth seems really unusual. Of note, we found one patient having a 5 cm lesion at the onset of HCC. The possible reason of a close HCC onset after SVR and the difference in lesion size could be related not only to immune system but also intrinsically correlated to HCC. Indeed previously has been demonstrated that HCC size could explain phenotypic diversity within proliferative and non-proliferative HCCs and be associated to vascular invasion according to dimension [32]. Therefore patients with largest tumor are correlated to different metabolic path of tumor with related poor prognosis [33].

In addition, one of the major bias of the study is the limited number of cases and the short follow-up time, but early HCC occurrence was the primary endpoint of the study and, for that reason, long term assessment was not part of this research protocol.

These results seem to be corroborated by the absence of any statistically significant difference in clinical and laboratory results between patients who underwent 2D–3D based treatment compared to other antiviral regimens, as well as in the use of ribavirin in CTP class A and B.

Nevertheless, during the follow-up of our patients the aim was mainly focused on the early appearance of HCC after treatment and, fascinatingly, this event occurred at all times of this short term follow-up period. Therefore, our findings significantly underline that we still have no large data on the possible effect of some DAAs treatment schedule on physiopathology of HCC natural history and that Ribavirin, probably due to its immunomodulatory properties, may represents a protective factor on HCC risk.

In these patients, others rigorous studies on molecular mechanisms and immune system on are necessary to better elucidate and understand subtended mechanisms of early HCC occurrence in this cohort. In particular, a limitation of this study is the lack of an external validation cohort for confirming our results.

## Conclusions

Early HCC occurrence appears to be associated with an advanced CTP stage, male gender, higher liver stiffness value and diabetes. However, interestingly, it is also more often correlated to SOF based therapy without Ribavirin, in CTP A, whereas the use of this latter antiviral seems exert a protective effect on HCC onset, as some literature also suggests [27, 28].

We therefore firmly believe that a careful selection during antiviral schedule evaluation, as well as follow-up, is mandatory, particularly in patients undergoing a SOF without RBV antiviral regimen.

## Data Availability

Data available if requested.

## Abbreviations

DAA: direct acting antiviral
HCV: hepatitis C virus
SVR: sustained virological response
HCC: hepatocellular carcinoma
TE: transient elastography
EASL: European Association for the Study of Liver
US: abdominal ultrasound
SIUMB: Italian Society of Ultrasound Medicine
CEUS: contrast-enhanced ultrasonography
CT: computed tomography
MRI: magnetic resonance imaging
CTP: Child–Turcotte–Pugh
BMI: body mass index
AIFA: Italian Medicines Agency Committee
SOF: Sofosbuvir
RBV: Ribavirin
BCLC: Barcelona Clinic Liver Cancer
